# Sensitization of cervix cancer cells to Adriamycin by Pentoxifylline induces an increase in apoptosis and decrease senescence

**DOI:** 10.1186/1476-4598-9-114

**Published:** 2010-05-19

**Authors:** Alejandro Bravo-Cuellar, Pablo C Ortiz-Lazareno, Jose M Lerma-Diaz, Jorge R Dominguez-Rodriguez, Luis F Jave-Suarez, Adriana Aguilar-Lemarroy, Susana del Toro-Arreola, Ruth de Celis-Carrillo, Jose E Sahagun-Flores, Javier E Garcia de Alba-Garcia, Georgina Hernandez-Flores

**Affiliations:** 1 División de Inmunología, Centro de Investigación Biomédica de Occidente, Instituto Mexicano del Seguro Social, Sierra Mojada 800, Colonia Independencia. Guadalajara, Jalisco, CP 44340, México; 2 Departamento Ciencias de la Salud, Centro Universitario de los Altos, Universidad de Guadalajara, Km 7,5 Carretera a Yahualica, Tepatitlán de Morelos, Jalisco, CP 47820, México; 3 Laboratorio de Inmunología, Centro Universitario Ciencias de la Salud, Universidad de Guadalajara, Sierra Mojada 950, puerta 7, Colonia Independencia. Guadalajara, Jalisco, CP 44340, México; 4 Unidad de Investigación Social, Epidemiológica y Servicios de Salud, Centro Médico Nacional de Occidente, Instituto Mexicano del Seguro Social, Belisario Domínguez 1000, Colonia Independencia, Guadalajara, Jalisco, CP 44340, México; 5 Hospital Regional Valentín Gómez Farías, Instituto de Seguridad Social y Servicios Sociales de los Trabajadores del Estado, Avenida Soledad Orozco 203, Colonia el Capullo, Guadalajara, Jalisco. CP 45150, México

## Abstract

**Background:**

Chemotherapeutic drugs like Adriamycin (ADR) induces apoptosis or senescence in cancer cells but these cells often develop resistance and generate responses of short duration or complete failure. The methylxantine drug Pentoxifylline (PTX) used routinely in the clinics setting for circulatory diseases has been recently described to have antitumor properties. We evaluated whether pretreatment with PTX modifies apoptosis and senescence induced by ADR in cervix cancer cells.

**Methods:**

HeLa (HPV 18+), SiHa (HPV 16+) cervix cancer cells and non-tumorigenic immortalized HaCaT cells (control) were treated with PTX, ADR or PTX + ADR. The cellular toxicity of PTX and survival fraction were determinated by WST-1 and clonogenic assay respectively. Apoptosis, caspase activation and ADR efflux rate were measured by flow cytometry, senescence by microscopy. IκBα and DNA fragmentation were determinated by ELISA. Proapoptotic, antiapoptotic and senescence genes, as well as HPV-E6/E7 mRNA expression, were detected by time real RT-PCR. p53 protein levels were assayed by Western blot.

**Results:**

PTX is toxic (WST-1), affects survival (clonogenic assay) and induces apoptosis in cervix cancer cells. Additionally, the combination of this drug with ADR diminished the survival fraction and significantly increased apoptosis of HeLa and SiHa cervix cancer cells. Treatments were less effective in HaCaT cells. We found caspase participation in the induction of apoptosis by PTX, ADR or its combination. Surprisingly, in spite of the antitumor activity displayed by PTX, our results indicate that methylxantine, *per se *does not induce senescence; however it inhibits senescence induced by ADR and at the same time increases apoptosis. PTX elevates IκBα levels. Such sensitization is achieved through the up-regulation of proapoptotic factors such as *caspase *and *bcl *family gene expression. PTX and PTX + ADR also decrease E6 and E7 expression in SiHa cells, but not in HeLa cells. p53 was detected only in SiHa cells treated with ADR.

**Conclusion:**

PTX is a good inducer of apoptosis but does not induce senescence. Furthermore, PTX reduced the ADR-induced senescence and increased apoptosis in cervix cancer cells.

## Background

Cervix cancer is the most frequently diagnosed female cancer in developing countries and the second most frequent cancer affecting women worldwide [1]. An estimate of half a million new cases in 2008 were reported [2]. The most important risk factor in this cancer is the presence of human papilloma virus (HPV) infection. High risk HPV types 16 and 18 are responsible for over 70% of cases of cervix cancer [3].

Cervix cancer, like other tumors shows two critical cellular stages: apoptosis and senescence. The first one occurs during normal or physiological conditions or by stimuli such as chemotherapy and constitutes a common pathway for cell replacement, tissue remodeling, damaged cell removal and elimination of cancer cells [4-6]. It is a complex process which involves caspases participation, activation of proapoptotic genes, among other molecules. Apoptosis is defined by morphologic features which include cell membrane blebbing, cell shrinkage, chromatin condensation, and nucleosomal fragmentation [7,8]. Cellular senescence, originally defined as a phenotype of arrested cells, after a certain number of cell divisions. Now is considered a general biological program of terminal growth arrest, and can be induced by the shortening of telomeres (growing old) or by injuries to DNA which do not involve telomere shortening (accelerated senescence) [9,10]. In this state, while they may remain metabolically active, cells can not divide even if they are stimulated by mitogens. They can be distinguished morphologically by their enlarged and flattened cell shape and increased granularity. This distinction is identifiable with considerable specificity by detection of β-galactosidase (SA-β-gal) by X-gal activity staining. Senescence shows a dual role in cancer patients. Since this process inhibits tumor cell proliferation it was considered to be a protection mechanism. However, recent data suggest that it also facilitates cancer progression [9-11].

Patients with advanced, persistent, or recurrent squamous cell carcinoma are usually treated with cytotoxic chemotherapeutic agents such as Adriamycin (ADR) which kills cancer cell mainly by apoptosis [9]. This drug can also induce senescence [12,13]; however, tumor cells can develop resistance to chemotherapy and generate responses of short duration or complete failure [14]. Molecular basis of resistance to cancer therapy is not well understood. It is considered that several factors can play a role. Among these mechanisms, the transcriptional nuclear factor-κB (NF-κB) is a key regulator of genes involved in cellular proliferation, secretion of soluble factors such as TNFα and up-regulation of antiapoptotic genes [15-19].

Pentoxifylline (PTX), [1-(5-oxohexyl) 3, 7,-dimethylxanthine], is a nonspecific phosphodiesterase inhibitor which has been already clinically and routinely used for circulatory diseases for more than twenty years, and it is a potent NF-κB and TNFα inhibitor. Recently, PTX has been used to sensitize tumor cells to chemo- and radio-therapy. In our experience, we have observed that lymphoma-bearing mice treated with PTX + ADR survived more than one year after receiving only half of the standard therapeutically active ADR dose [20,21]. PTX also sensitizes leukemic cells to perillyl alcohol-induced apoptosis [22] and also to prostatic tumor, HeLa and hepatoma cell line [23].

Cervix cancer is a public health problem and chemotherapy is not actually effective. Additionally, the effectiveness of chemotherapy has been limited by its side effects and/or the toxicity of these drugs in normal tissues and normal cells. Thus, the use of pharmacological agents that act by inhibiting resistance mechanisms or sensitizing cancer cells to chemotherapy without increasing side effects, are strongly desirable. We attempted to study *in vitro *whether pretreatment with PTX modifies apoptosis and senescence induced by ADR in cervix cancer cell lines.

## Results

### Cell viability of human cervix cancer cells after in vitro treatment with PTX or ADR either alone or in combination

We first evaluated cell sensitivity of HeLa, SiHa and HaCaT cells to *in vitro *treatment with either PTX alone or in combination with ADR, cell viability was determined in function of its metabolic activity by WST-1 assay. Table 1 shows the viability in comparison with untreated control cells (100%). Cells treated with PTX, had viability of 63.6 ± 2.1% in HeLa cells, 57.8 ± 1.0% in SiHa cells and 74.1 ± 1.0% in HaCaT cells. When we used ADR, the viability was 88.6 ± 3.0%, 70.1 ± 2.0%, and 95.0 ± 7.6% in HeLa, SiHa and HaCaT cells, respectively. Addition of ADR to cells pre-incubated with PTX, significantly decreased viability to 40.2 ± 1.0% for HeLa, 33.0 ± 1.2% for SiHa cells, but had little effect in HaCaT cells (78.5 ± 1.1% viability). These results strongly suggest that each drug individually decreases the viability of cervix cancer cells, but in combination these effects are increased.

**Table 1:** Viability of HeLa, SiHa or HaCaT cells after *in vitro *exposition to Pentoxifylline and Adriamycin

CELL LINE PERCENTAGE OF CELLULAR VIABILITY ± SD			
GROUP	HeLa	SiHa	HaCaT
**PTX 8 mM**	63.6 ± 2.1	57.8 ± 1.0	74.1 ± 1.0
**ADR 1 μM**	88.6 ± 3.0	70.1 ± 2.0	95.0 ± 7.6
**PTX + ADR**	40.2 ± 1.0	33.0 ± 1.2	78.5 ± 1.1

SD = standard deviation, Pentoxifylline = PTX, Adriamycin = ADR. Cultures cells were treated with PTX or ADR or their combination, 24 hours later the cells were harvested and cell viability was determined spectrophotometrically at 450 nm using WST-1 kit. The results are reported in percentage of cell viability as compared to untreated control groups respectively considered as 100% and the values represent the mean SD of three independent experiments.

### Clonogenic assay in HeLa, SiHa and HaCaT cells after in vitro treatment with PTX or ADR either alone or in combination

**Figure 1:**
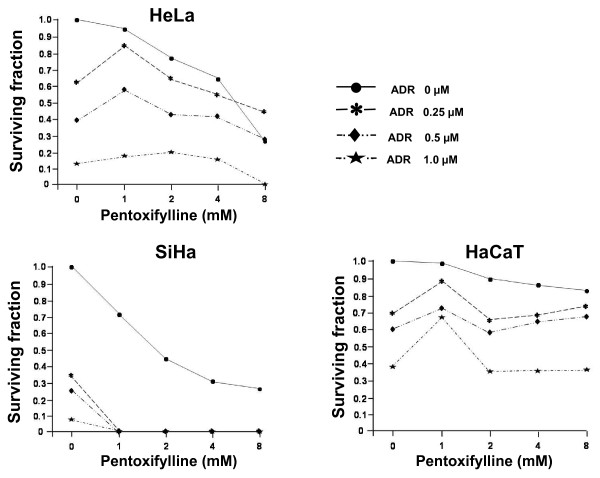
**Clonogenic assay of HeLa, SiHa and HaCaT cells *in vitro *exposure for 6 hours to Pentoxifylline (PTX 8 mM) or Adriamycin (ADR 1 μM) either alone or in combination**. The cells were washed, trypsinized and suspended in drug-free medium and plated in 6-well plates. After 12 days, the colonies were fixed stained and counted. The results are expressed as survival fraction and represent the mean. In all cases standard deviation was not greater than 0.03 of three independently experiments carrier out in triplicate. Statistical analysis Student "t" test.

The clonogenic assay is a proven method to study the toxicity of antitumor drugs. In Figure 1 we see when HeLa cells are incubated with PTX or ADR or their combination at different doses. The response was dose specific, showing little toxicity with low doses and incrementing progressively with higher doses. In fact, all groups show a linear regression comprise between r^2 ^= 0.86 and 0.98, *p *< 0.05. The most important toxicity in HeLa cells was reached with a dose of PTX (8 mM) + ADR (1 μM) which presented a survival fraction of 0.01 ± 0.03. This value is lower than the toxicities addition of each drug alone, (survival fraction, PTX 0.28 ± 0.04 and ADR 0.16 ± 0.03). For SiHa cells treated with PTX it was observed, than the dose-response effect was not as clear as in the previous experiment (r^2 ^= 0.69, *p <*0.16). In contrast, in SiHa cells exposed to ADR the dose-response effect was clear, reaching a survival fraction with a lower dose of ADR (0.25 μM) of 0.35 ± 0.03, and for the higher dose, the survival fraction decrease drastically 0.07 ± 0.02 (r^2 ^= 0.94, p < 0.03). Finally, SiHa cells were more sensitive to the drug combination, since the most important effect was also achieved detected when the cells were treated with PTX + ADR with the three different ADR doses. In the cloning assay, no cells were observed because it reached a 100% of toxicity. In the case of non-tumorigenic immortalized HaCaT cells it was not observed a dose-response effect and show a high degree of resistance in all cases (all groups r^2 ^= 0.38, *p *= 0.37). These results confirm our previous reports and suggest a probable potentializer effect between PTX and ADR.

### Pentoxifylline blocks Adriamycin efflux

**Figure 2:**
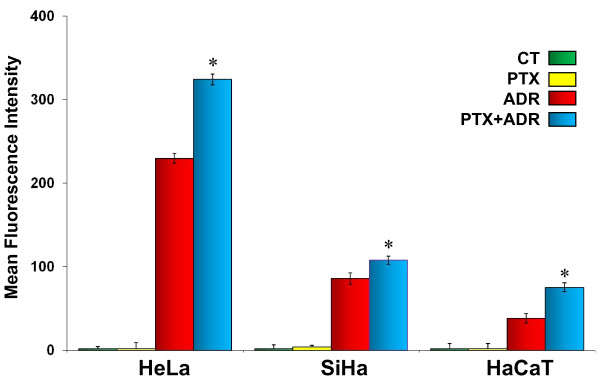
**Effect of Pentoxifylline in Adriamycin efflux**. ADR efflux was measured in HeLa, SiHa and HaCaT cells treated with PTX (8 mM), ADR (1 μM) or its combination and ADR efflux was determined by flow cytometry. The results represent the mean intensity fluorescence (MIF) ± standard deviation of three independent experiments carrier out in triplicate. Statistical analysis ANOVA test. (*) *p *< 0.05 *vs *ADR.

It's likely that PTX may have other effects than sensitize cancer cells to ADR such as reducing cellular ADR efflux. Based on the fact that ADR is fluorescent, we investigated the ADR efflux in HeLa, SiHa and HaCaT cells by flow cytometry. In Figure 2 we observed the ADR fluorescence in cervix cancer cells as well as in non-tumorigenic immortalized cells. This observation was most important in cancer cells; however the behavior was similar with three cell lines studied. The results show that the ADR concentration was higher in PTX + ADR group (MFI = HeLa 324.3 ± 5.8, SiHa 107.9 ± 6.7 and HaCaT 75.5 ± 5.4) than in the exclusively ADR group (MFI = HeLa 229.8 ± 6.3, SiHa 85.9 ± 4.7 and HaCaT 38.5 ± 5.4), *p *< 0.02. The results suggest that PTX inhibits ADR efflux helping to retain intracellular ADR.

### Early apoptosis induced by PTX alone or in combination with ADR

Early apoptosis was evaluated by three different methods, two of which were performed by flow cytometry: Annexin V stain test (based on phosphatidylserine determination) and the detection of H2A.X histone (DNA damage determination); the third method was an ELISA-based photometric assay that measures cytoplasmic DNA-histone nucleosome complexes generated during apoptotic DNA fragmentation. Table 2 shows a homogeneous response for HeLa cells among the three methods. In the untreated HeLa control group the percentage of apoptosis was 3.7 ± 1.0%, 2.7 ± 0.2% and 10.0 ± 1.5% for Annexin V, H2A.X histone and DNA-histone nucleosome tests respectively. Surprisingly, the presence of PTX in HeLa cell cultures induced apoptosis in an important way with values that ranged between 30.5 ± 1.1%, 28.7 ± 1.0% and 30.3 ± 2.3% for the three tests respectively (*p *< 0.001 in all cases *vs *corresponding untreated control cells). The exclusive presence of ADR in HeLa cell cultures also induced higher values of early apoptosis than untreated control cells; 23.3 ± 2.1%, 28.6 ± 1.8% and 20.0 ± 1.7% for Annexin V, H2A.X and DNA-histone nucleosome test respectively; *p *< 0.001 in comparison with the corresponding untreated control group. When the HeLa cells were incubated with PTX + ADR the most important values of early apoptosis were reached, 38.4 ± 1.6%, 43.3 ± 2.5% and 31.0 ± 1.5% for the three test respectively. These results are significantly higher than in the cells treated either with exclusively PTX or ADR alone (*p *< 0.001 with Annexin V and H2A.X assays); however for the DNA-histone nucleosome test the apoptosis was higher than the untreated control or ADR treated cells (*p *< 0.001) but similar to the exclusively PTX treated cells. The behavior of SiHa tumor cells was similar to that observed in the previous experiment. In the case of cell cultures treated with PTX + ADR, we observed the most significant values of early apoptosis 41.5 ± 1.0% (Annexin V), 45.4 ± 2.6% (H2A.X) and 30.5 ± 1.5% (DNA-fragments) which represent an increment over PTX-treated cells for each assay of 1.4, 2.4 and 1.5 times respectively (*p *< 0.005 in all cases *vs *corresponding cells treated only with PTX or ADR). The experiment was also carried out with immortalized non-tumorigenic cell (HaCaT). In this experiment we observed that when early apoptosis was measured by Annexin V staining, the most significant result was seen in the cultures treated with PTX + ADR (apoptosis = 23.1 ± 1.4%). A similar behavior was observed in ELISA test. When considering these results, it is important to stress that treatment with PTX + ADR induced a greater increase in apoptosis in HeLa and SiHa cells when compared with either PTX or ADR treatments as well as with HaCaT cells (*p *< 0.001).

**Table 2:** Early apoptosis of HeLa, SiHa or HaCaT cells

**HeLa**			
**GROUP**	**Annexin V**	**H2A.X**	**DNA-Fragmentation**
	**(% mean ± SD)**	**(% mean ± SD)**	**(% mean ± SD)**
CONTROL	3.7 ± 1.0	2.7 ± 0.2	10.0 ± 1.5
PTX 8 mM	30.5 ± 1.1 *	28.7 ± 1.0 *	30.3 ± 2.3 *
ADR 1 M	23.3 ± 2.1 *	28.6 ± 1.8 *	20.0 ± 1.7 *
PTX + ADR	38.4 ± 1.6 *	43.3 ± 2.5 *	31.0 ± 1.5 *
			
**SiHa**			
GROUP	Annexin V	H2A.X	DNA-Fragmentation
	(% mean ± SD)	(% mean ± SD)	(% mean ± SD)
CONTROL	3.7 ± 1.0	2.5 ± 1.0	10.0 ± 1.5
PTX 8 mM	28.4 ± 2.1 *	19.0 ± 1.2 *	20.5 ± 1.8 *
ADR 1 M	27.0 ± 1.5 *	33.0 ± 3.0 *	17.5 ± 2.7 *
PTX + ADR	41.5 ± 1.0 *	45.4 ± 2.6 *	30.5 ± 1.5 *
			
**HaCaT**			
GROUP	Annexin V	H2A.X	DNA-Fragmentation
	(% mean ± SD)	(% mean ± SD)	(% mean ± SD)
CONTROL	3.8 ± 0.2	3.3 ± 0.2	10.0 ± 1.5
PTX 8 mM	6.0 ± 0.7	6.2 ± 1.2	10.3 ± 1.8
ADR 1 M	15.5 ± 1.0 *	6.3 ± 0.9	10.2 ± 1.7
PTX + ADR	23.1 ± 1.4 *	5.5 ± 0.8	15.4 ± 1.5 *

Standard deviation = SD, Pentoxifylline = PTX, Adriamycin = ADR. Cell cultures were treated with PTX or ADR or their combination, 24 hours later the cells were harvested and apoptosis was determined by flow cytometry using Annexin V stained, H2A.X and DNA-histone nucleosome test by ELISA. The results represent the mean ± SD of three independent experiments carried out in triplicate. Statistical analysis Student "t" test. *p < 0.001 vs corresponding untreated control group.

### Late apoptosis induced by PTX, ADR or their combination

**Figure 3:**
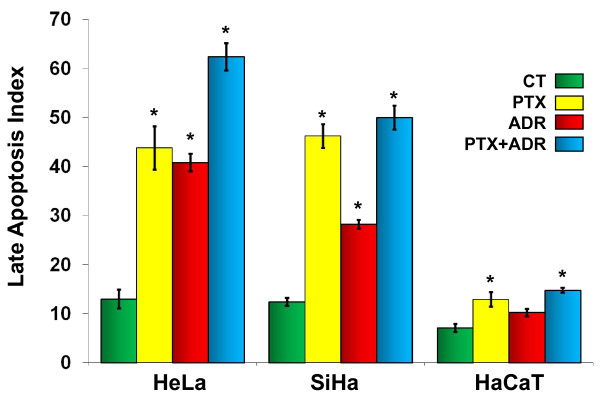
**Late apoptosis in HeLa, SiHa and HaCaT cells after *in vitro *exposure to Pentoxifylline (PTX) or Adriamycin (ADR) either alone or in combination**. Cell cultures were treated with PTX (8 mM) or ADR (1 μM) or their combination (PTX + ADR), 24 hours later the cells were harvested and apoptosis was determined by UV light microscopy using ethidium bromide and acridine orange stains. The results represent the percentage of apoptosis ± standard deviation of three independent experiments carrier out in triplicate. Statistical analysis Student "t" test. (*) *p *< 0.001 *vs *untreated cells (CT).

To confirm the preceding observations, we studied late apoptosis behavior in our experimental conditions by UV light microscopy using ethidium bromide and acridine orange. Figure 3 shows that the apoptotic indexes for untreated HeLa, SiHa and HaCaT cells were 13.0 ± 1.9, 12.4 ± 0.8 and 7.1 ± 0.8 respectively. When the cells were treated with PTX we observed apoptotic indexes of 43.4 ± 4.4, 46.2 ± 2.4 and 12.8 ± 1.5 for HeLa, SiHa and HaCaT cells respectively (*p *< 0.001 *vs *untreated cells). In HeLa, SiHa and HaCaT cell cultures treated with ADR, were observed 40.8 ± 1.8, 28.2 ± 0.9 and 10.2 ± 0.8 respectively (*p *< 0.001 comparing with untreated cells). When we used PTX + ADR we observed an increase in the apoptotic indexes of 62.4 ± 2.8, 50.0 ± 2.4 and 14.7 ± 0.5 in HeLa, SiHa and HaCaT cells respectively (*p *< 0.001 *vs *corresponding untreated cells). The whole apoptosis observations strongly suggest that PTX is a good inductor of apoptosis in cervix cancer cells and can also sensitize cancer cells to ADR-induced apoptosis.

### PTX and ADR induce caspase activation in HeLa and SiHa cells

**Figure 4:**
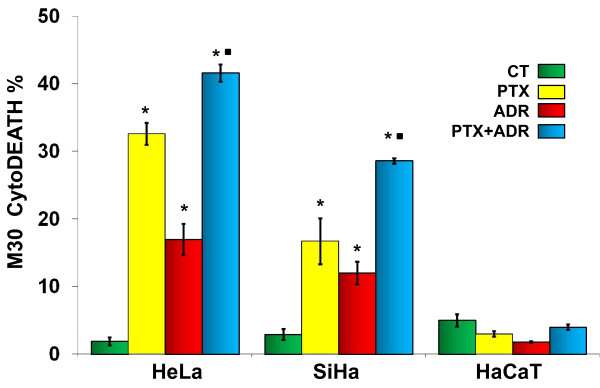
**Determination of caspases activity in HeLa, SiHa and HaCaT cells after *in vitro *treatment with Pentoxifylline (PTX) or Adriamycin (ADR) either alone or in combination**. Cell cultures were treated with PTX (8 mM) or ADR (1 μM) or their combination (PTX + ADR), 24 hours later the cells were harvested and caspase activity was determined measuring caspase-cleaved cytokeratin 18 neo-epitope M30 by flow cytometry (M30 CytoDeath™ Biotin Kit). The results represent the percentage of caspase activity ± standard deviation of three independent experiments carrier out in triplicate. Statistical analysis Student "t" test. (*) *p *< 0.001 *vs *untreated control cells (CT). (•) *p *< 0.001 *vs *PTX or ADR groups.

In order to study the participation of caspase-dependent apoptosis in our observations, we measured under the same experimental conditions as described above, the amount of the M30 epitope present on caspase-cleaved cytokeratin 18. Figure 4 shows that the percentage of M30 positive cells was minimum in all three cell lines without treatment with values between 1.9 ± 0.6% and 5.0 ± 0.9%. In HeLa and SiHa cells significant caspase activity was detected in all PTX and ADR treated cells; however, the most important results were observed principally in the PTX + ADR treated groups (*p *< 0.001 *vs *corresponding untreated control, PTX or ADR groups). In opposition to this, in HaCaT cells we did not observe caspase activity.

### PTX reduces ADR-induced senescence in cervix cancer cells

**Figure 5:**
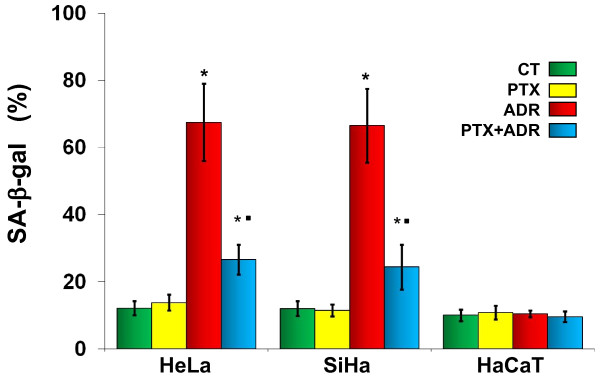
**Determination of SA-β-gal associated senescence of HeLa, SiHa and HaCaT cells after *in vitro *treatment with Pentoxifylline (PTX) or Adriamycin (ADR) either alone or in combination**. Cell cultures were treated with PTX (8 mM) or ADR (1 μM) or their combination (PTX + ADR), 24 hours later the cells were harvested and senescence was determined by histochemistry using the BioVision senescence detection kit. The results represent the percentage of SA-β-gal positive cells and represent the mean ± standard deviation of three independent experiments carrier out in triplicate. Statistical analysis Student "t" test. (*) *p *< 0.001 *vs *untreated control cells (CT). (•) *p *< 0.001 *vs *ADR.

Senescence is very relevant in Oncology and can be induced by chemotherapy. For this reason, we determined whether PTX or ADR alone or in combination induces senescence evaluating SA-β-Gal expression, a well established senescence marker. Figure 5 show that PTX in our experimental conditions does not induce senescence in HeLa and SiHa cells. However, as expected significant increases of senescence were observed in ADR treated HeLa (senescence = 67.5 ± 11.5%) and SiHa cells (senescence = 66.5 ± 11.0%), with values being 5.5 times higher than untreated control or PTX treated cells (*p *< 0.001). We also observed that PTX greatly reduces the senescence induced by ADR, since the percentage of senescence found in PTX + ADR treated cultures of HeLa cells (senescence = 26.6 ± 4.4%) and SiHa cells (senescence = 24.4 ± 6.7%) represents a diminution of 2.5 and 2.7 folds in relation to the ADR treated group (*p *< 0.001 for both groups). PTX, ADR or their combination did not induce senescence in HaCaT cells.

### PTX increases IκBα protein

**Figure 6:**
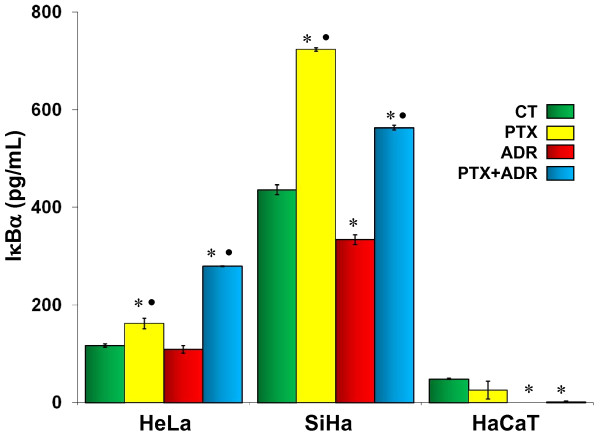
**Determination of IκBαin HeLa, SiHa and HaCaT cells after *in vitro *treatment with Pentoxifylline (PTX) or Adriamycin (ADR) either alone or in combination**. Cell cultures were treated with PTX (8 mM) or ADR (1 μM) or PTX + ADR, 24 hours later the cells were harvested, protein was extracted and IκBα was determined by ELISA assays. The results show pg/mL of IκBα and represent the mean ± standard deviation of three independent experiments carrier out in triplicate. Statistical analysis Student "t" test. (*) *p *< 0.005 *vs *untreated control cells (CT). (•) *p *< 0.005 *vs *ADR.

As NF-κB induces cell proliferation its inhibition could be part of the mechanism by which PTX and/or ADR works as chemotherapeutic drug. IκBα plays a central role in keeping NF-κB in the cytoplasm, and this blocks NF-κB activation. For this reason, we performed a set of experiments with ELISA test to determine whether PTX, ADR or their combination modify levels of cytoplasmatic IκBα. As Figure 6 shows, when HeLa and SiHa cells were treated with PTX, we observed IκBα accumulation (*p *< 0.005 *vs *ADR and respectively untreated control groups). In contrast, ADR treatment induced IκBα protein reduction which is more evident in SiHa and HaCaT cells (*p *< 0.005). Finally when the cervix cancer cells were treated with PTX + ADR we observed an increment of IκBα protein compared with untreated control and exclusively ADR treated cells (*p *< 0.005) and the contrary effect was observed in HaCaT cells.

### p53 protein expression and mRNA E6/E7 in HeLa and SiHa cells treated with PTX, ADR or PTX + ADR

**Figure 7:**
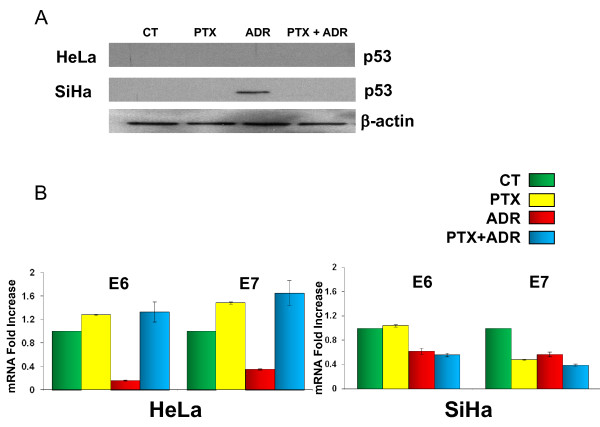
**Western blot detection of p53 protein and mRNA of E6 and E7 in HeLa and SiHa cells at 24 and 3 hours respectively after *in vitro *treatment with Pentoxifylline (PTX 8 mM) or Adriamycin (ADR 1 μM) either alone or in combination**. Effect of treatment on p53 protein, analysis by Western blot (a). Changes in the expression of E6 and E7 genes (b). Analysis of RT-PCR products was performed using LightCycler software. The data are expressed as fold increase or decrease of relative quantities, using ribosomal protein as a reference gene. The results represent the mean ± standard deviation of three independent experiments carrier out in triplicate.

It is well known that E6 and E7 oncogenes of HPV 16 and 18 are related to chemotherapy resistance and modulate negatively the expression of genes such as p53. For this, in our experimental conditions, we determined p53 protein and mRNA levels. The results in Figure 7a allow us to observe that p53 protein from HeLa cervical tumor cells, as expected was not found in the control group and the treated groups showed the same behavior. Results with SiHa cells are similar to those found with HeLa, however in this cell, ADR induces p53.

Oncoproteins E6 and E7 play a very important role because they show close relation with p53 regulation. For this reason were studied, in the same experimental conditions, the mRNA expression in both cervical tumor cell lines. In Figure 7b we can see that in HeLa cells, PTX increased the expression of E6 mRNA (Δ% = 28). The treatment with ADR induced a contrary effect in an important fashion (Δ% = -84) and the experimental group treated with PTX + ADR increased E6 mRNA expression in HeLa cells (HPV 18) with a Δ% = 33. In relationship to E7 we observed practically a mirror behavior of E6 in all treatments. In the same way we evaluated E6 and E7 in SiHa cells (HPV 16). PTX did not modify mRNA expression of E6 (Δ% = 4) with ADR or PTX + ADR was strictly similar between themselves and lower than the control group (Δ% = -38 and -44 respectively). For E7 from HPV 16 we observed an important down regulation being most important in PTX + ADR group (PTX Δ% = -52, ADR Δ% = -43, PTX + ADR Δ% = -61).

### mRNA expression of proapoptotic, antiapoptotic and senescence genes in cervix tumor cell lines after in vitro treatment with PTX alone or in combination with ADR

**Figure 8:**
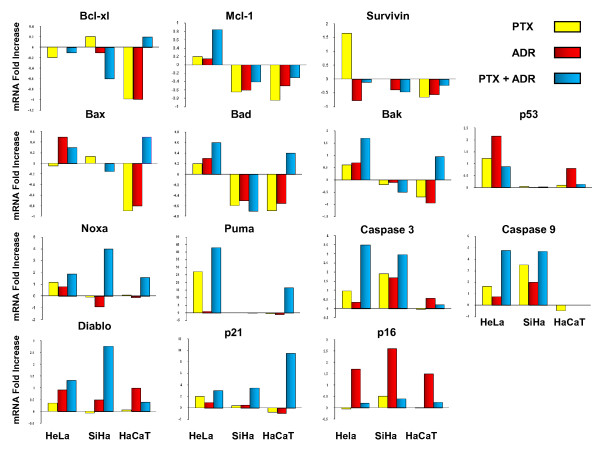
**Comparison of relative quantity of mRNA of proapoptotic, antiapoptotic and senescence related genes in HeLa, SiHa and HaCaT cells after *in vitro *exposure to Pentoxifylline (PTX) or Adriamycin (ADR)**. Measurements were conducted using real-time RT-PCR. Cells cultures were treated with PTX (8 mM) or ADR (1 μM) or their combination (PTX + ADR) for 3 hours. Analysis of RT-PCR products was performed using LightCycler software. The data are expressed as fold increase or decrease of relative quantities using ribosomal protein as a reference gene. Experiments were conducted in triplicates and repeated three times. In all cases standard deviation was not greater than 0.1.

We determined the effects of PTX, ADR or PTX + ADR on the expression of proapoptotic, antiapoptotic and senescence genes in HeLa, SiHa and HaCaT cells. Real-time PCR assay revealed that PTX treatment induced modifications in the proapoptotic gene expression. Figure 8 shows a 0.3 to 3 fold up-regulation in *bak, noxa, p21, p53, diablo*, c*aspase-3 *and *caspase-9 *genes mRNA expression levels in HeLa cell treated with PTX or ADR. In the same manner *puma *gene was up-regulated more than 25 fold following treatment. In addition, the ADR regulates others proapoptotic genes in HeLa cells. We observed 0.3 to 3 fold up-regulation of *bad *and *bax*, and senescence associated *p16 *gene mRNA expression levels on HeLa cells. In the same way, when the HeLa cells were treated with PTX + ADR, mRNA expression levels of *bad, bax, bak, noxa, p16, p53 *and *diablo *were 0.3 to 3 fold up-regulated, while in *p21, caspase-3 *and *caspase-9 *we found more than 3 fold up-regulation. However, we observed over 40 fold up-regulation in *puma *gene in this cell.

In SiHa cells treated with PTX, we found 0.3 to 3 fold up-regulation in *p16, p21*, *caspase-3*, moreover c*aspase-9 *was 4 fold up-regulated (Figure 8). When SiHa cells were treated with ADR we observed 0.3 to 3 fold up-regulation of *p21, diablo, caspase-3 *and *caspase-9, p16 *genes. In SiHa cells treated with PTX + ADR, we observed 0.3 to 3 fold up-regulation of *puma, diablo*, c*aspase-3 *and *p16*. However the up-regulation was greater in *caspase-9, p21 and noxa *more than 3.7-fold increase (Figure 8). We also found up-regulation in *bad, bax, bak, noxa, puma, p21*, and *diablo *gene in HaCaT cells treated with PTX + ADR. It was especially significant in *puma *and *p21 *genes being above a 10 fold increase. On the contrary antiapoptotic genes *bcl-_XL_, mcl-1*, and *survivin *were down-regulated or not modified in practically all cellular lines by the different treatments, with the exception of *survivin *gene in HeLa cells (1.6 fold increase) treated with PTX and *mcl-1 *in HeLa cells (0.8 fold increase) treated with PTX + ADR. These data suggested the up-regulation in favour of genes with proapoptotic activity principally in HeLa and SiHa cancer cells treated with PTX + ADR.

## Discussion

This work shows that PTX *per se *may induce apoptosis of cervix cancer cell lines but does not induce senescence. In the same manner, but no less important, PTX sensitizes these cells to the induction of apoptosis by ADR and at the same time reduces significantly the senescence caused by this anthracycline.

Infections with high-risk HPV are the predominant associated risk factors in the development of cervical cancer. In addition, proteins E6 and E7 expressed by HPV contribute to resistance to treatments [23]. We used HeLa and SiHa cells infected with HPV 18 and 16 respectively to be closer to the most common HPV high risk types observed in patients.

The studies of toxicity in cervix tumor cells, including clonogenic assay show that PTX has antitumoral activity by itself and the most important results are observed when the cells are pretreated with PTX before ADR. All these results strongly suggest that the combination of PTX + ADR not only induced death in tumor cells, but also reduced the number of cells with reproductive capacity after treatment.

Early and late apoptosis were also induced more efficiently by the combination of the drugs in the two cervix cancer cell lines. Our results are in agreement with previous experiments and with the published data [24], suggesting that the sensitizing effect of the PTX is due to a general mechanism, present in different cell lines and not in a particular one, even if the cells are infected with high risk HPV.

To explain the differences in late apoptosis in HeLa and SiHa culture treated with ADR, it is important consider that late apoptosis it is not reversible and each system could differ depending the genetic background of the cells. For example HeLa cells have 50 integrated copies of the HPV 18 whereas SiHa cells have only two copies of the HPV 16 and its rate of replication is very different [25].

The effect of PTX alone or in combination with ADR has been related to caspase activation [21,26]. At this point it is necessary to stress that in general our results are in agreement with other experiments reported here, so the higher or lower activity of caspases was correlated with higher or lower percentage of apoptosis.

Senescence originally was defined as cell arrest growth [10], and considered a novel tumor suppressive mechanism [27]. However, the role of senescence in Oncology has to be enlightened. It is true that the senescent cells can not replicate itself, but it can stimulate the growth and dissemination of other tumor cells by secretion of different products like growth factors, cytokines and metalloproteases. It has been reported that senescent cells, induce an increment in the cell proliferation of precancerous cells [28,29]. To our knowledge, this is the first report indicating that PTX alone can induce apoptosis but does not induce a senescence cell state. In addition, it is also very important to remark that PTX pretreatment significantly reduces ADR-induced senescence and in turn increases the apoptotic index in HeLa and SiHa cancer cells. Our observations support also the idea that independently of the type of HPV virus that infects these cells, they can undergo apoptosis which is preferable, to cellular senescence.

These results can be explained biochemical and biologically by different facts: PTX is an effective inhibitor of phosphodiesterase (PD) activity (mainly PD4), and has also been reported to prevent the arrival of the NF-κB to the cellular nucleus by inhibition of the phosphorylation of serine 32 and 36 of the IκB complex in lymphoma and the human monocyte cell line U937 [21,24]. In this sense, we find that PTX alone or in combination with ADR causes an increment of IκBα in HeLa and SiHa cells. This accumulation of IκBα induced by PTX could lead to a significant retention and therefore less activation of NF-κB, which in turn suppresses survival signals induced by this factor.

Our results show that PTX decreases ADR efflux in HeLa and SiHa cells and this increases the ADR cytotoxic effect. It has been reported that PTX down-regulated *mdr1 *gene expression and avoid ADR efflux, and in turn, therefore decrease the resistance of L1210 leukemic cells to ADR effects [30].

This methylxanthine arrests the cell cycle in the G2 phase, in which tumor cells are more sensitive to the toxic effects of some chemotherapeutic agents and radiotherapy [22,31]. ADR can induce reactive oxygen species which are described as apoptosis or senescence inductors [32]. PTX has been described as an antioxidant molecule [33] and this may explain why PTX alone does not induce senescence but impairs the senescence induced by ADR.

Using Real-Time PCR assay, we studied the mRNA expression of genes related with induction of apoptosis, caspases and senescence. In general we observed an up-regulation of proapoptotic genes. The most important up-regulation is observed in *caspase-3 and caspase-9, noxa, puma*, and *diablo *and down-regulation in antiapoptotic family genes in HeLa and SiHa cells treated with PTX, ADR or both.

We observed up-regulation in *p53 *gene in HeLa cells and in some groups of SiHa cells. The p53 protein was not found except in SiHa cells treated exclusively with ADR. This could indicate that apoptosis by PTX is p53-independent and suggests the participation of other molecules such as caspase-3 and caspase-9 as observed in this study. The up-regulation of the *puma *gene and p53 protein abrogation in these cells treated with PTX lets us to suppose that multiples pathways may have been involved to induce puma, such as endoplasmic reticulum stress, FOXO3a and p73 [34].

We determined p53 protein levels in HeLa and SiHa cells treated with PTX, ADR and PTX + ADR and its relationship with HPV E6 and E7 genes from HPV 18 and 16 respectively. Interestingly, ADR induced p53 protein stabilization in SiHa cells and reduced mRNA levels of HPV E6 and E7 in both cancer cells. In this sense, besides the fact that ADR did not modify the mRNA levels of p53 in SiHa cells, it impaired the degradation that could lead to growth arrest or apoptosis. If survival factors are also induced then growth arrest is favoured and the cell goes in senescence. Premature senescence has been described to be associated with increases in p53, p21, and p16 levels [35,36].

When we measured the levels of mRNA expression of HPV E6 and E7 in PTX + ADR treated cells we observe a down-regulation in SiHa and an opposite effect in HeLa cells. In this respect we can only speculate that the transcriptional machinery used by HPV 16 and 18 is modulated differentially by PTX.

It has been described that up-regulation of p16 is associated with senescence [35,36]. We detected up-regulation of *p16 *gene when SiHa and HeLa cells were treated with ADR and this is in agreement with the senescence observed in these cells and contrarily when the cells were treated with PTX.

## Conclusion

Our observations show antitumor activity of PTX. This molecule induces apoptosis in a p53 independent manner and decreases the senescence induced by ADR. Importantly, PTX increases the sensitivity of cancer cells to the toxic effects of ADR by caspase participation and regulation of *caspase -3*, *caspase-9*, *puma*, *noxa*, and *diablo *genes.

## Methods

### Cell lines

HeLa (HPV 18+) and SiHa (HPV 16+) cervical cancer cell lines and the spontaneously immortalized human epithelial cell line HaCaT (used as non-tumorigenic control cells) were kindly provided by Dr. Boukamp (DKFZ-Heidelberg, Germany). All the cell lines were maintained *in vitro *and propagated in Dulbecco's modified Eagle's culture medium (DMEM) supplemented with 10% heat-inactivated fetal bovine serum, 1× L-glutamine (2 mM final concentration) and antibiotics (penicillin/streptomycin), (this medium will be referred to as DMEM-S), and incubated at 37°C in an humidified atmosphere containing 95% air and 5% CO_2_. All the previously mentioned products were obtained from GIBCO™ Invitrogen Corporation (Carlsbad, CA, USA).

### Drugs and experimental conditions

Adriamycin (ADR) was obtained from Lemery Laboratories, México, and stocked at 4°C less than 4 days and adjusted to desirable concentration with DMEM culture medium immediately before utilization. Pentoxifylline (PTX) (Sigma, Saint Louis MO, USA) dissolved in sterile saline solution 0.15 M, at a concentration of 0.2 M and kept at 4°C less than 4 days.

### Cell culture and in vitro treatments

HeLa, SiHa and HaCaT cells suspended in DMEM-S at concentrations of 1.5 × 10^6 ^or 2 × 10^6^/8 mL in exponential phase were seeded in p100 Petri dishes for flow cytometry assays, senescence and Western blot studies. For survival test and for apoptosis determined by ELISA, the cells were cultured in 96-well plates at a concentration of 3 × 10^5 ^cells/well/200 μL (final volume). For clonogenic assays the cells were seeded at densities of 1 × 10^4 ^cells/2 mL in 6-well plates. In all cases the cells were cultured overnight at 37°C in a humidified atmosphere containing 5% of CO_2 _and 95% air. The medium was then replaced with DMEM-S. Then the cells were either treated with PTX 8 mM, or ADR 1 μM or PTX + ADR (final concentrations). The cells were incubated with PTX 1 hour before ADR addition and 24 hours later the culture cells were harvested. In the cases for genes expression study the cells were incubated with the drugs for only 3 hours.

### Cell survival

Cells survival was determined by the cleavage of tetrazolium salt WST-1 into formazan by cellular mitochondrial dehydrogenase enzyme. 24 hours after each treatment the cell were incubated as above indicated with 10 μL/well of WST-1/ECS solution (BioVision Research, Mountain View, CA, USA) and incubated for 3 hours in the same conditions. The absorbance (450 nm) of the treated and untreated samples was determined on a microtiter plate reader (Synergy™ HT Multi-Mode Microplate Reader. Biotek Winooski, VT, USA). Data are reported in percentage of cell survival as compared to respectively untreated control group considering as 100%.

### Clonogenic Survival Assays

Cells were assayed for the cytotoxic effects of PTX or ADR or PTX + ADR after cell survival according to the established methods of performing the clonogenic assay [37]. Subconfluent cultures were exposed to drugs for 6 hours. Then the cells were washed with PBS preheated to 37°C, trypsinized and plated in 6-well plates (100 cells/wells). After 12 days of incubation in complete culture medium, the colonies were stained with crystal violet after fixation with formaldehyde and counted manually. In each case the results are expressed as survival fraction which was obtained by dividing the number of colonies after the treatment by the number of cells seeding and the results multiply by the plate efficiency (PE). PE = (N°. of colonies formed/N° of cells seeded) ×100.

### Adriamycin efflux assay

HeLa, SiHa and HaCaT cells were incubated with ADR for 1 hour in DMEM-S at 37°C. For ADR efflux measurement, the cells were washed with DMEM-S and treated or untreated with PTX for 2 hours at 37°C. The cells were washed, trypsinized and collected in drug-free medium. ADR fluorescence was determined in FACSAria cell sorter (BD, Bioscience, San Jose, CA, USA). For tests at least 20,000 events were analyzed for each sample. Data were processed with the FACSDiva software package (BD). The results are reported as the mean intensity fluorescence.

### Apoptosis and caspase activity detection methods

Cellular detection of Annexin V, H2A.X phosphorylation and M30 caspase activity were determined by flow cytometry using the fluorescein isothiocyanate conjugated monoclonal Annexin V-FITC apoptosis Kit (Annexin-V-Fluos; Roche, Mannheim, Germany), H2A.X phosphorylation assay kit (H2A.X Phosphorylation Assay Kit, Millipore Temecula, CA, USA) and M30 CytoDEATH™ Biotin antibody (Roche Mannheim, Germany) respectively according to the manufacturer's instructions. For the three tests at least 20,000 events were analysed for each sample in an EPICS XL-MCL™ Flow Cytometer Beckman Coulter model (Fullerton, CA, USA). Data were processed with the System II software package (Beckman Coulter).

### Apoptosis Detection by ELISA

In normal untreated and treated cell cultures we determinated cytoplasmic histone-associated-DNA-fragments (mono- and oligonucleosomes) spectrophotometrically (420 nm) using Cell Death Detection Elisa^PLUS ^(Roche Mannheim, Germany) according the manufacturer's instruction [38]. The enrichment of mono- and oligonucleosomes released into the cytoplasm was calculated: experimental absorbance/corresponding control absorbance. The results are expressed as the percentage of DNA fragmentation.

### Acridine orange/ethidium bromide staining to detect late apoptosis by UV-microscopy

Briefly the cells were stained with ethidium bromide (Sigma Saint Louis MO, USA) and acridine orange (Sigma, Saint Louis MO, USA) (100 μg/mL each). Two hundred cells were counted and number of each of the following 4 cellular states recorded: i) live cells with normal nuclei (LN), bright green chromatin and organized structure; ii) apoptotic cells (A) with highly condensed or fragmented bright green-yellow chromatin; iii) dead cells with normal nuclei (DN), bright red chromatin and organized structure and iv) dead cells with apoptotic nuclei (DA) and bright orange chromatin, which were highly condensed and fragmented. Apoptotic index (AI): A + DA/LN +A +DN +DA × 100.

### β-galactosidase associated senescence

According to the manufacturer's instructions senescence was determined by histochemical in treated and untreated control cells by Senescence Detection Kit (BioVision Mountain View, CA, USA) which detects β-galactosidase activity (SA-β-gal) present in senescence cells. We counted 300 cells of six microscopic fields to determine the percentage of SA-β-gal stained positive cells identified by an intense blue stain in the membrane.

### Protein extraction for IκBα ELISA and p53 western blot assay

1-2 × 10^6 ^cells were seeded in p100 culture Petri-dishes and treated next day with PTX, ADR and PTX + ADR for 24 hours. After treatment, cells were harvested by scraping and lysed with RIPA buffer (0.5% deoxycholate, 0.5% NP-40, 0.5% SDS, 50 mM Tris pH 7.4 and 100 mM NaCl) containing protein inhibitors. Following sonication (15 pulses, 50% amp), protein extracts were obtained after 30 min incubation at 4°C and 5 min centrifugation at 14 000 rpm/4°C. Protein concentrations were determined using BioRad Dc Protein Kit.

### IκBα detection

The levels of IκBα protein was determined in HeLa, SiHa and HaCaT treated and untreated control cells using a commercial ELISA kit at 450 nm (Invitrogen), according to the manufacturer's instructions. The results are expressed in pg/mL.

### p53 Western blot analyses

Total cell protein (50 μg) was subjected to electrophoresis using a 12% sodium dodecyl sulfate polyacrylamide gel. Subsequently, proteins were transferred to Immobilon-P PVDF membranes (Millipore) and incubated with 1× western blocking reagent (Roche) during 1 hour for nonspecific binding. Immunodetection of p53 was performed using anti-p53 (DO-1, dilution 1:750, Santa Cruz biotechnology, Inc.) at room temperature for 2 hours. After incubation with a horseradish peroxidase-conjugated secondary antibody, immunoreactive proteins were visualized by western blotting luminol reagent in chemiluminiscence films and resolved on BioMax film (Kodak) according to the manufacture's specifications. Control β-actin antibody.

### Quantitative real time PCR

Total RNA from both cells was obtained after 3 hours of incubation using the PureLink™ Micro-to-Midi total RNA purification system (Invitrogen Corporation, Carlsbad, CA, USA). First-strand cDNA was synthesized from 5 μg of total RNA using Superscript™ III First-Strand Synthesis Supermix (Invitrogen Corporation, Carlsbad, CA, USA). Real Time PCR was performed using a LightCycler^® ^2.0 apparatus (Roche Applied Science, Mannheim, Germany) and LightCycler-FastStart DNA Master^PLUS ^SYBR Green I (Roche Applied Science, Mannheim, Germany). Analysis of PCR products was performed using LightCycler^® ^software (Roche Applied Science, Mannheim, Germany). The data are expressed as relative quantities using a reference gene (Protein Ribosomal). Each sample was processed in triplicate to verify the specificity of the amplification reaction. Oligonucleotides (Invitrogen Corporation, Carlsbad, CA, USA) used to amplify human *bad*, *bax, bak, noxa, puma, diablo, caspase-3, caspase-9, mcl-1, survivin, bcl-_XL_, p21, p53*, *p16, E6 and E7 (HPV16 and HPV18) *and Ribosomal Protein are shown in Table 3. Oligonucleotides were designed using the Oligo v.6 software. Gene sequences were obtained from the GenBank Nucleotide Database of the National Center for Biotechnology Information http://www.ncbi.nlm.nih.gov.

**Table 3:** Primer pairs used for real-time quantitative PCR

Gene	Primer pair sequences
*bad*	5'CTC CGG AGG ATG AGT GAC GAGT 3'
	5'ACT TCC GCC CAT ATT CAA GAT 3'
*bak*	5'CGC TTC GTG GTC GAC TTC AT 3'
	5'AGA AGG CAA AGA CTT CGC TTA 3'
*bax*	5'TTT GCT TCA GGG TTT CAT CC 3'
	5'CAG TTG AAG TTG CCG TCA GA 3'
*noxa*	5'GAG ATG CCT GGG AAG AAG G 3'
	5'TCC TGA GCA GAA GAG TTT GGA 3'
*puma*	5' GAT GGC GGA CGA CCT CAA C 3'
	5'TGG GAG TCC AGT ATG CTA CAT GGT 3'
*survivin*	5'TGA GCT GCA GGT TCC TTA TCT G 3'
	5'GAA TGG CTT TGT GCT TAG TTT T 3'
*diablo*	5'TGA CTT CAA AAC ACC AAG AGT A 3'
	5'TTT CTG ACG GAG CTC TTC TA 3'
*p21*	5'CGA CTT TGT CAC CGA GAC AC 3'
	5'CGT TTT CGA CCC TGA GAG T 3'
*p53*	5'CTG AGG TTG GCT CTG ACT GTA CCA CCA TCC 3'
	5'CTC ATT CAG CTC TCG GAA CAT CTC GAA GCG 3'
*p16*	5'CAG TAA CCA TGC CCG CAT AGA T 3'
	5'TGA AAA GGC AGA AGC GGT GT 3'
*mcl-1*	5'CAC GAG ACG GTC TTC CAA GGA TGC T 3'
	5'CTA GGT TGC TAG GGT GCA ACT CTA GGA 3'
*bcl-XL*	5'GCA GGC GAC GAG TTT GAA CT 3'
	5'GTG TCT GGT CAT TTC CGA CTG A 3'
*Caspase-3*	5'ATA CTC CAC AGC ACC TGG TTA T 3'
	5'AAT GAG AGG GAA ATA CAG TAC CAA 3'
*Caspase-9*	5'GTA CGT TGA GAC CCT GGA CGA C 3'
	5'GCT GCT AAG AGC CTG TCT GTC ACT 3'
*E6 (HPV 18)*	5'GCG ACC CTA CAA GCT ACC TGA T 3' 5'GCA CCG CAG GCA CCT TAT TA 3'
*E7 (HPV 18)*	5'TGT CAC GAG CAA TTA AGC GAC T 3' 5'CAC ACAAAG GAC AGG GTG TTC A 3'
*E6 (HPV 16)*	5'CAG AGC TGC AAA CAA CTA TAC 3' 5'AGT GGC TTT TGA CAG TTA ATA C3
*E7 (HPV 16)*	5'GAC AAG CAG AAC CGG ACA G 3' 5'ATT CCT AGT GTG CCC ATT AAC A 3'
*ribosomal*	5'GCA TTG ACA ACA GGG TTC GTA G 3'
*protein*	5'ATT TAA ACA GAA AAC GTG CAC A 3'

Oligonucleotides were designed using the Oligo v.6 software. Gene sequences were obtained from the GenBank Nucleotide Database of the National Center for Biotechnology Information http://www.ncbi.nlm.nih.gov.

### Statistical analysis

Results of each experiment represent mean ± standard deviation (SD) of 3 independent experiments carried out in triplicate. Differences between groups were determined by Student "t" test and ANOVA. Significant results were considered with *p *< 0.05. For the comparison of gene expression, it was only considered positive or negative differences ≥30% in relationship to reference values. In some cases was calculated the Δ% that represent the percent of increment or diminution in relationship to comparative group.

## Competing interests

The authors declare that they have no competing interests.

## Authors' contributions

ABC, GHF, JMLD, JRDR, RCC and JESF carried out experimental work. AAL, LFJS and STA performed molecular study. ABC, PCOL, JMLD, JEGAG and GHF performed the statistical analysis, conceived and drafted the manuscript. All authors helped to draft the manuscript and approved this final version.
